# Leakage? or Secretion? unconventional protein secretion in cancer

**DOI:** 10.18632/oncotarget.28368

**Published:** 2023-02-20

**Authors:** Kohji Yamada, Kiyotsugu Yoshida

**Keywords:** unconventional protein secretion, cancer, endoplasmic reticulum

Extracellular soluble proteins, which lack typical N-terminal signal peptides, were reported around 30 years back [1]. Many eukaryotic secretory proteins penetrate the endoplasmic reticulum (ER) and are secreted via the classical secretory vesicle-related pathway mediated by the Golgi apparatus. Importantly, in this classical pathway, proteins have N-terminal signal peptides that permit their migration from the cytoplasm into the ER via the translocon. In contrast, the secretion of proteins that do not code for signal peptides may occur via two or more mechanisms, often collectively referred to as unconventional protein secretion (UPS).

Tumors are frequently exposed to harsh environmental conditions, and membrane disruption in tumor cells is particularly likely to occur. Therefore, even if the active release of cytosolic proteins, namely UPS, occurs, it is quite difficult to determine whether cytosolic proteins detected in the extracellular space are derived from leakage or UPS. Various leaked cytosolic proteins are present in the bloodstream of cancer patients. Therefore, in clinical settings, cellular damage is an indicator of chronic hepatitis, and blood marker tests are conducted. Particularly, in liver cancer, cytosolic proteins are secreted actively, but not just leaked. In our experiments with liver cancer cell lines, we demonstrated that proliferating cells under normal culture conditions release importin α1 and PKCδ [2, 3]. Notably, PKCδ secretion was markedly inhibited when intracellular PKCδ was activated by phorbol 12-myristate 13-acetate treatment [3]. Thus, we showed that the inactive form of PKCδ is actively secreted extracellularly. The serum levels of PKCδ were shown to be significantly higher in patients with hepatocellular carcinoma than those in healthy subjects, patients with chronic hepatitis, and patients with cirrhosis [3]. The fact that the markers of liver damage are elevated in chronic hepatitis indicates that PKCδ release is not correlated with cellular injury. In addition, serum PKCδ levels have been found to be elevated in patients with early-stage cancers [4]. Therefore, UPS occurs clinically in cancer, indicating its potential application as a biomarker.

Several reports have described the extracellular localization of cytosolic proteins in cancer. In many cases, they are involved as humoral factors in tumor promotion. Eustace et al. found that HSP90α is secreted extracellularly and is related to the invasiveness of fibrosarcoma and breast cancer cells [5]. Compared with healthy individuals, high levels of plasma HSP90α have been detected in patients with breast, lung, pancreatic, or liver cancer [6]. Christian et al. demonstrated that nucleolin is localized on the cell surface of neuroblastoma tumor cells and endothelial cells in angiogenic tumor blood vessels [7]. Recently, we found that PKCδ interacts with Glypican 3, a heparan sulfate proteoglycan, at the cell surface and subsequently enhances tumor growth by activating IGF1R-ERK1/2 signaling. *In vivo* studies have also shown that tumor growth is markedly suppressed by anti-PKCδ antibodies. A series of studies have indicated that UPS occurs in cancer cells.

In addition to its contribution to tumorigenesis, several mechanistic features of UPS in cancer are evident. For instance, UPS in cancer occurs in a stimulus-independent manner. In contrast, UPS of many well-known proteins, including IL-1β and HMGB1, is triggered by stimuli such as proinflammatory cytokines. In liver cancer cell lines, PKCδ and importin α1 were detectable in culture supernatants under normal culture conditions (10% FBS-containing medium). Furthermore, although most of the UPS is known to occur in a Golgi reassembly-stacking protein (GRASP)-dependent manner, PKCδ secretion is apparently independent of GRASP. In addition, our recent study showed that the interaction of PKCδ or importin α1, and nucleolin with E-Syt1 is essential for their secretion. E-Syt1 is a transmembrane protein localized in the ER, indicating that the ER may be involved in cancer-related UPS. Electron microscopy showed entrapment of PKCδ into SEC22B-positive vesicles. On the other hand, knockdown of ATG5, ATG7, and SEC22B caused suppression of PKCδ, importin α1, and nucleolin secretion, analogous to other well-known UPS, such as secretion of IL-1β, suggesting a relationship with autophagy.

In conclusion, we and other researchers have accumulated a series of evidences on UPS and its biological significance in cancer. However, the mechanism of action of UPS in cancer cells remains unclear. How cytosolic proteins localized to the ER via E-Syt1 migrate into vesicles for secretion or whether their secretory mechanisms involve other organelles, including the Golgi apparatus, autophagosomes, or lysosomes, remains unclear. In the future, if the route of UPS that is centered on E-Syt1 is elucidated, this will presumably lead to not only a basic understanding of liver cancer but also to the development of diagnostic and therapeutic strategies.
